# The association between angiotensin receptor blockers and lung, bladder, and colon cancer development: A 10-year multicentric retrospective Lebanese study

**DOI:** 10.1097/MD.0000000000034901

**Published:** 2023-09-08

**Authors:** Yara G. Dagher, Sandra El Helou, Karen G. Haifa, Issam G. Chalhoub, Rita T. Boulos, Bachir Atallah, Fadi Nasr, Issam Kassab, Mirna N. Chahine

**Affiliations:** a Faculty of Medical Sciences, Lebanese University, Hadath, Lebanon; b Statistics, Lebanese University, Hadath, Lebanon; c Hematology-Oncology Department, Hotel Dieu DE France, Achrafieh, Beirut, Lebanon; d National Center of Pharmacovigilance, Faculty of Pharmacy, Lebanese University, Hadath, Lebanon; e Basic Sciences Department, Faculty of Medical Sciences, Lebanese University, Hadath, Lebanon; f Foundation-Medical Research Institutes (F-MRI), Beirut, Lebanon/Geneva, Switzerland.

**Keywords:** ARBs, bladder, cancer, cardiovascular diseases, colorectal, hypertension, Lebanon, lung

## Abstract

Cardiovascular diseases (CVD) are the leading cause of death globally, followed by cancer. Angiotensin II contributes greatly to CVD pathogenesis, and Angiotensin II receptor blockers (ARBs) constitute a mainstay in hypertension and CVD management. However, the relationship between ARBs and cancer initiation is controversial, with no clear data in Lebanon. Therefore, our study aimed to determine the association between ARBs intake and lung, bladder, and colorectal cancers development in the Lebanese population. A retrospective study was conducted on 709 subjects divided into 2 main groups: Control (subjects without cancer; n = 177), and Cases (patients with cancer (n = 532): lung, bladder, or colorectal), taking ARBs (n = 236, (n = 121 in control and n = 115 in cases)) or not (n = 473). Collected information included the patients demographics, comorbidities, cancer’s risk factors, and ARBs dose and duration intake. Bivariate, multivariate, and binary logistic analyses were enrolled. ARBs use was significantly protective (*P* value = 0.000) against overall cancer development (odds ratio [OR] = 0.127) and against each, lung (OR < 1), bladder (OR < 1), and colorectal cancers (OR < 1). A duration-response relationship was established. This protective effect and the time-dependent relationship remained unchanged after omitting the most relevant risk factors. In summary, a significant overall protective effect of ARBs against lung, bladder and colorectal cancers was found. This beneficial response was time-dependent. These results can guide patients on treatment options and clinicians for informed decision-making.

## 1. Introduction

Cardiovascular diseases (CVD), which include disorders affecting the heart and blood vessels such as coronary artery disease (CAD), cerebrovascular accidents, peripheral arterial disease, heart failure, etc, are the leading cause of death globally, responsible for approximately 1-3rd of all deaths; predominantly occurring in developing countries.^[1]^

Systemic arterial hypertension (HTN) was found to be an independent risk factor for CVD development, and its treatment has been proven to reduce CVD-related mortality.^[1]^ The renin-angiotensin-aldosterone system (RAAS), which plays a primary role in the pathogenesis of essential HTN,^[2]^ is activated in volume-depleted states. Angiotensin II (Ang II), the major mediator of RAAS,^[3]^ increases water reabsorption in the kidneys and induces vasoconstriction, thus increasing blood pressure.^[4]^ Sustained RAAS activation will lead to the development of HTN^[5]^ which will cause endothelial dysfunction by creating an imbalance between relaxant (mainly nitric oxide) and contractile (mainly Ang II) endothelial factors. Consequently, a deleterious synergistic endothelial effect consists of diffuse vascular remodeling, collagen synthesis by vascular smooth muscles, fibrosis, and proinflammatory and proatherogenic changes.^[4]^

Antihypertensive treatment mainly includes diuretics, beta-blockers, calcium channel blockers, and RAAS inhibitors [angiotensin-converting enzyme inhibitors (ACEIs) and angiotensin II receptor blockers (ARBs)].^[6]^ RAAS inhibitors, considered a first-line treatment of essential HTN,^[4]^ are among the most efficient drug classes in preventing cardiovascular events.^[6]^ ARBs are favored over ACEIs since they are less likely to cause cough, angioedema, pancreatitis, and GI bleeding.^[7,8]^

The ARBs act by selectively blocking the angiotensin type 1 receptor (AT_1_R) on vascular smooth muscles and in the kidneys^[9]^ and thus prevent vasoconstriction, salt-water retention, aldosterone secretion, and the endothelial proinflammatory effect. The selective blockade of AT_1_R will lead to an indirect stimulation of angiotensin type 2 receptor (AT_2_R)^[3]^ and increase the metabolism of Ang II into Angiotensin 1 to 7, both inducing vasodilation, natriuresis, and endothelial antiproliferative actions.^[4]^ Both molecular and cellular evidence were found indicating that the expression and actions of the RAAS influence malignancy and also predict that RAAS inhibitors can play a role in cancer pathogenesis.^[7]^

Cancer is the second leading cause of death after heart disease and stroke,^[10]^ leading to 10 million deaths in 2020.^[11]^ According to the World Health Organization, 19.3 million new cancer diagnosis were made in 2020.^[12]^ In Lebanon, the incidence of cancer has been increasing by 4% to 5% annually since 2008,^[13]^ reaching 11,600 new cancer diagnosis in 2020.^[12]^ In our study, we discussed 3 of the most common types of cancer worldwide and in Lebanon specifically: lung cancer, bladder cancer, and colorectal cancer (CRC). Lung cancer is the leading cause of cancer-related death globally,^[14]^ followed by CRC. The latter is the third most common cancer diagnosis globally, while bladder cancer is among the most diagnosed cancers in men.^[14]^ These cancer incidences and mortality rates are expected to rise over the next twenty years.^[12]^

The association between ARBs and cancer is controversial. While some studies demonstrated a decreased risk for overall cancer associated with ARBs use and this mainly due to blocking the AT1R effect,^[15]^ others showed its use increased the incidence of some neoplasms (carcinogenic compounds of some ARBs: N-nitrosodimethylamine in valsartan in 2018 followed by N-nitrosodiethylamine in valsartan, losartan and irbesartan, and third nitrosamine product later on,^[16]^ and also the unopposed action of the AT_2_R, etc.).^[17]^ Moreover, a cohort study showed a neutral effect of ARBs use on lung and CRC.^[18]^

Despite this disputable cause-effect relationship and the lack of data concerning this association in Lebanon, we designed a retrospective study to determine the association between ARBs intake and the risk of cancer development in Lebanese healthy subjects or hypertensive patients. We also assessed if this relationship was dependent of the dose and duration of ARBs treatment, correlated with any cancer type and its associated risk factors, the gender and age of the patient, the associated comorbidities (CAD, HTN, and diabetes mellitus [DM]) and their duration, and other medications use.

### 1.1. Methods

#### 1.1.1. Ethical aspects.

This study was conducted in a manner that warranted the confidentiality of all included patients. The study had no potential risks since it was extracted from patients medical files during their hospital stay.

Data collection was obtained after approval from the thesis committee of the Faculty of Medical Sciences at the Lebanese University and the institutional review board committee of Lebanese Hospital Geitaoui (2021-IRB-010) and Mount Lebanon Hospital (Onc-2021-002). This study was conducted in accordance with Good Clinical Practice ICH Section 3, and the principles laid down by the 18th World Medical Assembly (Helsinki, 1964) and all applicable amendments. Moreover, patients were reached by phone to fill in the missing data from the medical files. Phone calls were done based on an informed consent. Finally, precautions were taken to keep personal identifier data separate from data collected.

#### 1.1.2. Study design and population.

A multicentric, retrospective, case-control study was conducted on Lebanese healthy subjects (control) or patients with comorbidities enrolled in 2 Lebanese hospitals affiliated to the Lebanese University, Faculty of Medical Sciences: Lebanese Geitaoui Hospital and Mount Lebanon Hospital.

The minimum sample size was calculated using G-Power, with an effect size of 0.5, an alpha error of 5%, and a power of 95%. A total number of 339 was obtained, divided into 2 main groups: control (n = 135; subjects with no cancer +/− ARBs) and cases (n = 204; subjects diagnosed with lung (n = 68), bladder (n = 68), or colorectal cancer (n = 68) between 2011 and 2021 +/− ARBs.) Therefore, a total of 4 subgroups were obtained: cancer-free patients/No ARBs use (group 1), lung, bladder or CRC patients/No ARBs use (group 2), cancer-free patients/ARBs use (group 3), lung cancer, bladder cancer or CRC patients/ARBs use (group 4).

The study included: Lebanese citizens aged between 30 and 100, diagnosed or not with any of the following cancers (bladder, colon, and lung cancers, between 2011 and 2021), Lebanese healthy subjects or hypertensive patients, with or without ARBs use where ARBs therapy was initiated at least 12 months before the cancer diagnosis.

The study excluded subjects with primary diagnosis of any cancer before 12 months of ARBs therapy, age below 30 or above 100, and subjects with insufficient history in the database.

#### 1.1.3. Data Collection: questionnaires and surveys.

The data was retrieved from the hospital’s medical files. Collection of data started in October 2019 and ended in August 2021. For each patient, questions were filled on a google form sheet.

The questionnaire included the following parameters: Patient demographics (year of birth, gender, socioeconomic status), characteristics (height, weight, BMI, smoking status), comorbidities (CAD, HTN, DM, dyslipidemia, chronic kidney disease), cancer status (no cancer, lung cancer, bladder cancer, colon cancer), cancer risk factors, and intake status of ARB (name, dosage, and duration of use).

#### 1.1.4. Data Analyses.

Data was analyzed using IBM SPSS version 25. Bivariate analysis was enrolled to test the association between ARBs use (all ARBs and each molecule alone), and overall cancer development, while controlling comorbid factors (CVD, HTN, DM, dyslipidemia, and their duration, gender, age smoking status, BMI and other medications use). Tests used were Chi-square test, Fisher exact test, and Mann–Whitney *U* test. Bivariate analysis was also enrolled in order to test the association between ARBs use and the development of each cancer type. In addition, using the multivariate analysis, we aimed to assess the association between the use of other medications (Proton pump inhibitors (PPI), metformin, aspirin, ACEIs, and fenofibrates) on cancer development, as these were the most encountered medications. Binary logistic analysis was performed to predict factors affecting each of the 3 cancer types where odds ratio (OR) with 95% confidence intervals was presented. Statistical significance was indicated at the 0.05 level.

### 1.2. Data availability

The data associated with the paper are not publicly available but are available from the corresponding author on reasonable request.

## 2. Results

### 2.1. Demographics

Our study included 709 patients meeting the required inclusion criteria. Among them, 63% were males, more than 60% were once smokers, the mean BMI was 26.9 kg/m^2^ and the mean age was 66.5 years. Patients were classified between control groups (25%) and cancer groups (75%), the latter divided between lung cancer, bladder cancer, and CRC. (Table 1)

**Table 1:** Baseline characteristics of the study population.

			Frequency	Percent
Gender	Male		448	63.2
	Female		261	36.8
Age	N		709	
	Mean (SD)		66.5 (11.5)	
	Median [Min - Max]		67 [27–98]	
BMI	N		596	
	Mean (SD)		26.9 (4.9)	
	Median [Min - Max]		26.2 [15.42–51.42]	
Smoking status	Non smoker		257	36.2
	Ex-smoker		84	11.8
	Current smoker		368	51.9
	Total		709	100.0
Cardiovascular disease	No		518	73.1
	Yes		191	26.9
	Mean (years): 8.8			
	Total		709	100.0
Diabetes	No		507	71.5
	Yes		202	28.5
	Mean (yr): 11.7			
	Total		709	100.0
Dyslipidemia	No		488	68.8
	Yes		221	31.2
	Mean (yr): 10.2			
	Total		709	100.0
Hypertension	No		284	40.1
	Yes		425	59.9
	Mean (yr): 11.4			
	Total		709	100.0
Group	Control		178	25.1
	Cancer	Total	531	74.9
		Lung	171	24.1
		Bladder	171	24.1
		Colorectal	189	26.7
	Total		709	100

Data are mean (SD) [Minimum-Maximum] for quantitative variables or percent for categorical.

BMI = body mass index (Kg/m^2^), SD = standard deviation.

Among our study population, 33.4% were on ARBs, with a duration ranging from 1 to more than 10 years. Four ARBs molecules were essentially used: Candesartan (9.4%), Valsartan (7.5%), Irbesartan (7.1%), and Telmisartan (6.9%). The usage of Losartan, Azilsartan, and Olmesartan was negligible, thus, they were dropped from our study. To assess if the relation between ARBs and cancer development was affected by the patient’s past medical history, the prevalence of each of CAD, DM, and HTN was studied. Moreover, cardiovascular patients are known to have several other comorbidities and will be on many other medications, hence, we included the most encountered medications: aspirin, PPIs, and metformin.

### 2.2. ARBs and cancer development

ARBs users represented 21.7% and 68.5% of cancer patients and control patients, respectively. Our study showed that ARBs use was significantly protective against overall cancer development. It is worth mentioning that this effect was also significant with the use of each molecule in particular, such as in the case of Candesartan, Valsartan, Telmisartan, and Irbesartan. (Table 2)

**Table 2:** ARBs effect on cancer development.

		Group		Total	*P* value	OR	95% Confidence interval	
		Control	Cancer				Lower	Upper
ARBs use	No	56	416	472	.000	0.127	0.087	0.185
		31.5%	78.3%	66.6%				
	Yes	122	115	237				
		68.5%	21.7%	33.4%				
Candesartan (atacand)	No	142	500	642	.000	0.245	0.146	0.409
		79.8%	94.2%	90.6%				
	Yes	36	31	67				
		20.2%	5.8%	9.4%				
Valsartan (diovan, entresto)	No	154	502	656	.000	0.371	0.210	0.656
		86.5%	94.5%	92.5%				
	Yes	24	29	53				
		13.5%	5.5%	7.5%				
Telmisartan (micardis)	No	150	510	660	.000	0.221	0.122	0.400
		84.3%	96.0%	93.1%				
	Yes	28	21	49				
		15.7%	4.0%	6.9%				
Irbesartan (avapro)	No	149	510	659	.000	0.212	0.117	0.382
		83.7%	96.0%	92.9%				
	Yes	29	21	50				
		16.3%	4.0%	7.1%				

ARBs = angiotensin II receptor blockers, OR = odds ratio.

By using the bivariate analysis, we found that ARBs were protective against lung cancer, bladder cancer, and CRC. This effect was found to be time-dependent following the multivariate analysis. The increased duration of ARBs use was associated with higher protection from each cancer development. (Table 3).

**Table 3:** Association between the duration of ARBs use with each of lung cancer (A), bladder cancer (B), and CRC (C).

A-lung cancer	B	S.E.	Wald	df	Sig.	OR	95% CI for EXP (B)	
							Lower	Upper
Duration of use of Candesartan (yr)	−0.185	0.051	13.028	1	0.000	0.831	0.752	0.919
Duration of use of Valsartan (years)	−0.116	0.053	4.772	1	0.029	0.890	0.802	0.988
Duration of use of Telmisartan (yr)	−0.156	0.056	7.843	1	0.005	0.855	0.767	0.954
Duration of use of Irbesartan (yr)	−0.221	0.054	16.488	1	0.000	0.802	0.721	0.892

B-Bladder cancer	B	S.E.	Wald	df	Sig.	OR	95% CI for EXP (B)	
							Lower	Upper
Duration of use of Candesartan (yr)	−0.147	0.048	9.457	1	0.002	0.863	0.785	0.948
Duration of use of Valsartan (yr)	−0.175	0.070	6.181	1	0.013	0.839	0.731	0.964
Duration of use of Telmisartan (yr)	−0.146	0.064	5.193	1	0.023	0.864	0.763	0.980
Duration of use of Irbesartan (yr)	−0.233	0.068	11.698	1	0.001	0.792	0.693	0.905

C-colorectal cancer	B	S.E.	Wald	df	Sig.	OR	95% CI for EXP (B)	
							Lower	Upper
Duration of use of Candesartan (yr)	−0.160	0.055	8.297	1	0.004	0.852	0.764	0.950
Duration of use of Valsartan (yr)	−0.203	0.073	7.738	1	0.005	0.816	0.708	0.942
Duration of use of Telmisartan (yr)	−0.138	0.068	4.082	1	0.043	0.871	0.762	0.996
Duration of use of Irbesartan (yr)	−0.225	0.074	9.191	1	0.002	0.799	0.690	0.924

ARBs = angiotensin II receptor blockers, CRC = colorectal cancer, OR = odds ratio.

### 2.3. The effect of cancer-specific risk factors and comorbidities on lung cancer, bladder cancer and CRC development

Lung cancer was more prominent in males (*P* = .001). Following the multivariate analysis to assess for all the potential confounding factors (cancer-specific risk factors (Table 4), comorbidities (CAD, HTN, and DM), and other medications use), PPIs and smoking were the only 2 factors significantly affecting our results, showing a strong positive correlation (harmful association) with lung cancer development with an OR of 3.45 and 10.52 respectively. (Table 5)

**Table 4:** Risk factors of lung cancer, bladder cancer, and CRC.

Lung cancer				Control	Lung cancer		Total	*P* value
Gender		Male		94	119		213	.001
				52.8%	69.6%		61.0%	
		Female		84	52		136	
				47.2%	30.4%		39.0%	
Risk factors		Group	Total			*P* value	OR (95% confidence interval)	
Smoking		No	86	16	102	.000	9.114 (5.039–16.484)	
			48.3%	9.3%	29.1%			
		Yes	92	156	248			
			51.7%	90.7%	70.9%			
Pollution including asbestos exposure		No	20	17	37	.695	1.147 (0.579–2.272)	
			11.2%	9.9%	10.6%			
		Yes	158	154	312			
			88.8%	90.1%	89.4%			
Family history of lung cancer		No	178	170	348	.149	0.489 (0.439–0.544)	
			100.0%	98.8%	99.4%			
		Yes	0	2	2			

CRC		Control	Cancer	Total	*P* value		OR (95% confidence interval)	
Age more than 50	No	22	19	41	.472		1.269 (0.662–2.434)	
		12.4%	10.0%	11.1%				
	Yes	156	171	327				
		87.6%	90.0%	88.9%				
Personal history of IBD, colorectal cancer and or polyps	No	175	179	354	.088		3.259 (0.882–12.041)	
		98.3%	94.7%	96.5%				
	Yes	3	10	13				
		1.7%	5.3%	3.5%				
Family history of colorectal cancer	No	177	174	351	.001		15.259 (1.994–116.766)	
		99.4%	92.1%	95.6%				
	Yes	1	15	16				
		0.6%	7.9%	4.4%				
Smoking	No	86	102	188	.279		0.797 (0.529–1.202)	
		48.3%	54.0%	51.2%				
	Yes	92	87	179				
		51.7%	46.0%	48.8%				
Alcohol	No	157	172	329	.378		0.739 (0.376–1.451)	
		88.2%	91.0%	89.6%				
	Yes	21	17	38				
		11.8%	9.0%	10.4%				
Diet: red meat, unhealthy food	No	49	48	97	.644		1.116 (0.701–1.775)	
		27.5%	25.4%	26.4%				
	Yes	129	141	270				
		72.5%	74.6%	73.6%				
Overweight	No	49	49	98	.132		0.689 (0.424–1.120)	
		29.2%	37.4%	32.8%				
	Yes	119	82	201				
		70.8%	62.6%	67.2%				
Physical inactivity	No	51	72	123	.055		0.653 (0.421–1.011)	
		28.7%	38.1%	33.5%				
	Yes	127	117	244				
		71.3%	61.9%	66.5%				
Bladder cancer		Control	Cancer	Total	*P* value		OR (95% confidence interval)	
Age more than 50	No	22	13	35	.134		1.725 (0.839–3.545)	
		12.4%	7.6%	10.0%				
	Yes	156	159	315				
		87.6%	92.4%	90.0%				
Male	No	84	41	125	.000		2.855 (1.806–4.513)	
		47.2%	23.8%	35.7%				
	Yes	94	131	225				
		52.8%	76.2%	64.3%				
Personal history of chronic bladder infection and or stones or polyps	No	166	154	320	.213		1.617 (0.754–3.466)	
		93.3%	89.5%	91.4%				
	Yes	12	18	30				
		6.7%	10.5%	8.6%				
Family history of bladder cancer	No	177	168	345	.208		4.214 (0.466–38.089)	
		99.4%	97.7%	98.6%				
	Yes	1	4	5				
		0.6%	2.3%	1.4%				
Smoking	No	86	55	141	.002		1.989 (1.287–3.072)	
		48.3%	32.0%	40.3%				
	Yes	92	117	209				
		51.7%	68.0%	59.7%				
Occupational exposure (aniline dyes, leather, rubber and paint)	No	175	169	344	1.000		1.036 (0.206–5.202)	
		98.3%	98.3%	98.3%				
	Yes	3	3	6				
		1.7%	1.7%	1.7%				
Pelvic irradiation	No	176	168	344	.442		2.095 (0.379–11.590)	
		98.9%	97.7%	98.3%				
	Yes	2	4	6				
		1.1%	2.3%	1.7%				
OR, odds ratio								

CRC = colorectal cancer, OR = odds ratio.

**Table 5:** Factors significantly affecting lung cancer (A), bladder cancer (B) and CRC (C).

(A) Lung cancer	B	S.E.	Wald	Df	Sig.	OR	95% CI for EXP (B)	
							Lower	Upper
PPI	1.237	0.302	16.747	1	0.000	3.447	1.906	6.234
Smoking	2.354	0.337	48.877	1	0.000	10.525	5.440	20.361

(B) Bladder cancer	B	S.E.	Wald	Df	Sig.	OR	95% CI for EXP (B)	
							Lower	Upper
Gender	−1.143	0.272	17.670	1	0.000	0.317	0.187	0.543
Diabetes	−0.838	0.281	8.877	1	0.003	0.469	0.249	0.751
Hypertension	−1.003	0.299	11.268	1	0.001	0.457	0.204	0.659
Smoking	0.585	0.261	5.022	1	0.025	1.794	1.076	2.992
Age (cutoff 55)	1.213	0.347	12.250	1	0.000	3.365	1.706	6.638

(C) CRC	B	S.E.	Wald	df	Sig.	OR	95% CI for EXP (B)	
							Lower	Upper
Hypertension	−0.681	0.324	4.418	1	0.036	0.506	0.268	0.955
PPI	−0.961	0.437	4.840	1	0.028	0.383	0.163	0.900
Family history of colorectal cancer	2.529	1.097	5.318	1	0.021	12.541	1.462	107.598
Age (cutoff 50)	1.355	0.471	8.280	1	0.004	3.875	1.540	9.750

CRC = colorectal cancer, OR = odds ratio, PPI = proton pump inhibitors.

Male gender was associated with an increased risk of bladder cancer (OR = 3.15). After adjustment for potential confounders, we found that the female gender, having DM and HTN were all protective factors against tumor enhancement showing a negative correlation with an OR of 0.32, 0.47, 0.46 respectively. On the contrary, smoking and age above 55 years were shown to increase the risk of bladder cancer with an OR of 1.79 and 3.37 respectively. (Table 5)

As opposed to lung and bladder cancer, there was no male predominance among CRC patients. When using the multivariate analysis, PPIs and hypertension were found to be protective against CRC development with an OR of 0.38 and 0.51, respectively. Family history of CRC and age above 50 years were all associated with an increased risk of malignancy with an OR of 12.54 and 3.87 respectively. (Table 5)

## 3. Discussion

**Figure 1.**
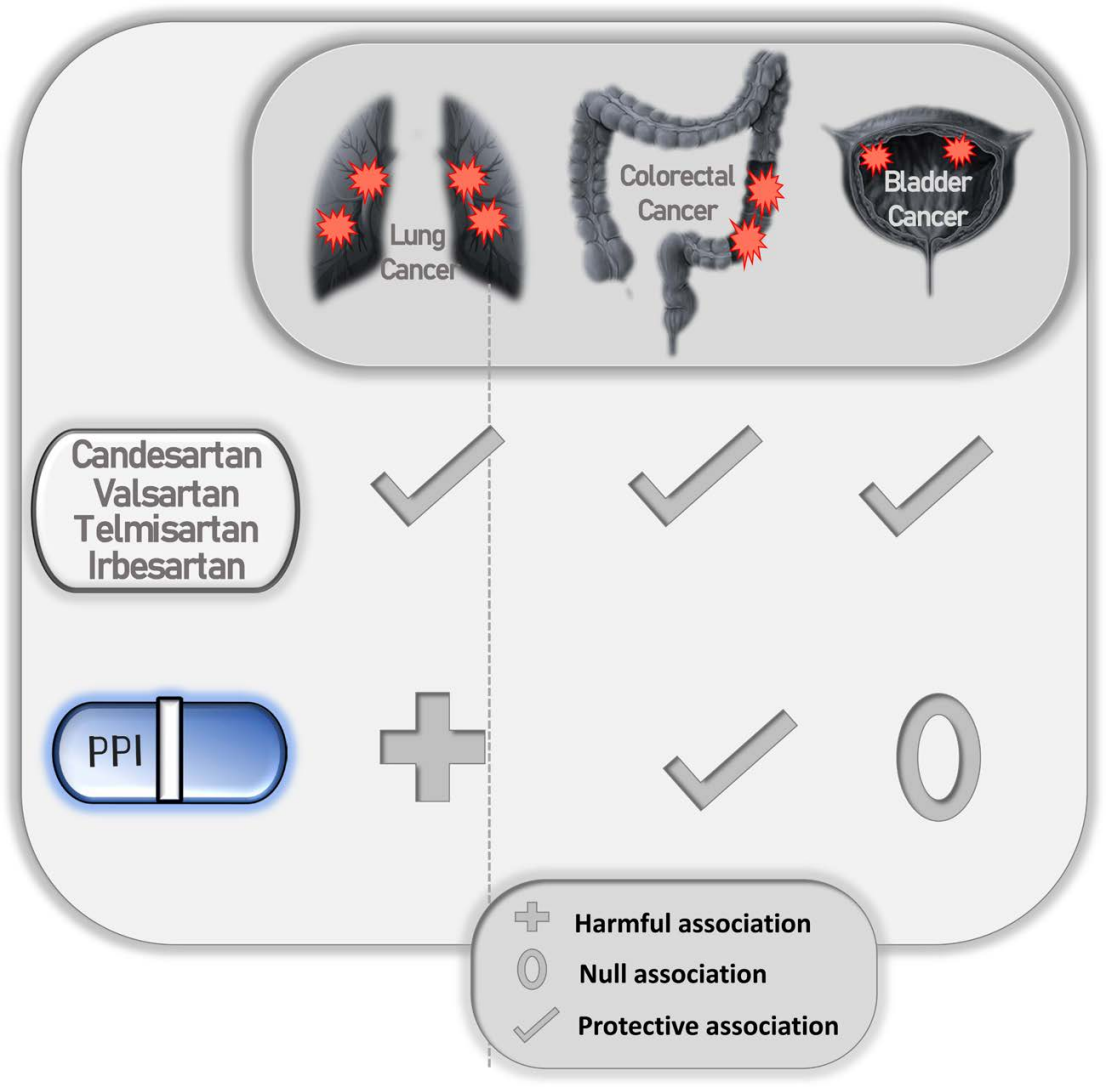
Protective vs Harmful association between different medications use such as ARBs, PPIs, and the incidence of lung, colorectal, and bladder cancer. ARBs = angiotensin II receptor blockers, PPI = proton pump inhibitors.

We used retrospective data to evaluate the impact of ARBs on the long-term risk for the development of lung, bladder, and CRC. We found important evidence of a decreased overall risk of cancer diagnosis associated with ever using an ARB. This protective effect persisted following subtype stratification in all cancer types, demographic adjustments, and through the years of drug use. It is also worth mentioning that after omitting the most relevant risk factors, including smoking in lung and bladder cancer, age in bladder and CRC, and family history and physical inactivity in CRC, this effect remained unchanged. In addition, PPIs showed a harmful association with lung and bladder cancers which contrasts to their protective association with CRC development. A summary of ARBs and PPIs toward cancer development is shown in Figure 1.

Our study findings are in contrast with some cases in the literature supporting the fact that ARBs use increases cancer development. For instance, the CHARM study found that Candesartan increases cancer deaths compared with the placebo group in patients with heart failure. In addition, in the LIFE study, lung cancer was significantly increased among ARBs users compared to the placebo group (relative risk (RR) = 2.41). The combination of Ramipril and Telmisartan showed a significant increment in cancer risk with a RR of 1.4 in the ONTARGET study. In the TRANSCEND study, there was a significant increase of 24% (RR = 1.24) in malignancy development among patients receiving Telmisartan compared to placebo-treated patients.^[10]^ Finally, Sipahi et al^[19]^ stated in their meta-analysis a modest increase in lung cancer risk with a RR of 1.25.

Other studies reported a neutral relationship between ARBs consumption and cancer development.^[18]^

In contrast to all the aforementioned studies, a protective effect of ARBs against tumor enhancement has been proved in several analyses. Gowtham Adamane et al proclaimed in their study carried out on over 1 million patients that ARBs treatment was protective against lung cancer [hazard ratio = 0.74 (0.67–0.83, *P* < .0001)], and this effect was most prominent after the 3^rd^ year of treatment initiation.^[20]^ Furthermore, Sungji et al outcomes were also consistent with our findings and showed an inverse association between ARBs medication and lung cancer development, and that relation persisted after stratified analysis according to age, gender, chronic obstructive pulmonary disease, never smokers, and never drinkers, but without significant dose nor duration-response relationship.^[21]^ A study conducted in Japan proved that inhibition of RAAS, for instance by administering ARBs, had a major effect on impeding non-muscle invasive bladder cancer recurrence.^[22]^ Furthermore, ARBs were considered an independent prognostic factor for overall survival and cancer-specific survival for bladder cancer patients post radical cystectomy.^[23]^

The reason behind these controversial results across the different studies could be attributed to the not yet clarified role of AT_2_R in cellular proliferation and migration.^[17]^ Another hypothesis suggested by a trial level analysis indicates that risk of cancer increases with increasing cumulative exposure to ARBs which started at 3 years of exposure in general and 2.5 years in lung cancer.^[16]^

Our study provided evidence that ARBs users did not have any additional risk of developing CRC, rather, ARBs showed to be protective.^[24–26]^

Furthermore, after performing a multivariate analysis and eliminating the effect of risk factors, our study validated the preventive effect of ARBs on overall cancer risk. These findings are in line with other studies that suggested that the protective effect of ARBs persisted when adjusted to different risk factors.^[20,21]^

### 3.1. Potential mechanisms of action of ARBs protective effect

The association between overall cancer risk and ARBs remains controversial and unclear. As previously mentioned, ARBs exert their effect on cancer development by blocking AT_1_R, leaving AT_2_R unopposed, with a concomitant increase in Ang^[1–7]^ leading to antiangiogenesis, anti-proliferation, and systemic vasodilatation^[6]^ thus eventually reaching an anti-inflammatory state, which explains the possible mechanism by which ARBs reduce cancer development risk.

RAAS inhibition was also mentioned as a possible target for immunotherapy in several cancer types; in a matter of fact, AT_1_R provokes hypoxia in tumor cells, and increases inflammatory cytokines and reactive oxygen species; all of which can lead to an immunosuppressive environment.^[27]^ Therefore, by using immunotherapy to target those pathways, we can improve therapy by improving medication distribution to the tissues and lowering required drug doses without compromising efficacy.^[27]^

### 3.2. Limitations of our study

Our study has several limitations: First, this was a retrospective study where most of the data were collected from medical files and completed by interviewing the patients by phone; therefore, recall bias and missing data were a concern in our study.

Second, our sample size was relatively small. This study included 709 patients, and this number made it hard for us to further subclassify the variables. For instance, analysis of the possible relation between cancer development and each type of ARBs was not feasible. This limitation might be crucial since few ARBs types were previously linked with cancer development.^[28]^

Third, a dose-response relationship was also difficult to establish. Since each of the types (Candesartan, Irbesartan, Valsartan, Losartan, Telmisartan) have different pharmacodynamics, and due to the restricted sample size of each 1, therefore a causality was not attainable neither in general nor by type.

Last but not least, we could not enroll all the important medications in our study. Limiting factors were the small sample size in ACEIs and fenofibrate, and the different types of molecules, with different mechanisms of action in diuretics.

### 3.3. Strengths of our study

In contrast, our study has also various strengths. First, despite being a global topic of concern, there is no data about the effect of ARBs use on cancer development in Lebanon, and our study is the first to address it. Second, our research is a multicentric study in which 709 patients were selected over a 2-year period from the Lebanese population, which includes about 6 million Lebanese citizens,^[29]^ in contrast to other studies conducted on thousands of patients in much larger populations.^[29]^ Therefore, our sample size of 709 patients is appropriate, relative to the small Lebanese population sample size. Therefore, Lebanese physicians can benefit from our findings and should take them into account while prescribing ARBs.

## 4. Conclusion

In summary, in this retrospective study, we found a significant overall protective effect of ARBs against lung cancer, bladder cancer, and colorectal cancer, and these findings remained evident following risk factors- stratified analysis. In addition, the beneficial response of AT_1_R blocking was time-dependent: the longer the treatment period, the smaller the chance of developing cancer. Despite the relatively small sample size, this study may be considered a reference for physicians when prescribing an antihypertensive drug. This is a lifelong treatment, and awareness must be present about any possible late side effects. We hope that our study will pave the way for more research on this topic, particularly to establish a clearer link between the putative protective or harmful effects of ARBs on cancer risk, as it may influence many cancer patients decisions making.

## Acknowledgments

We are mostly grateful to Lebanese Geitaoui Hospital (LGH) and Mount Lebanon Hospital (MLH) who provided us with their list of patients. We thank the patients who kindly accepted to respond to our questions when data were missing from their files.

## Author contributions

**Conceptualization:** Sandra El. Helou, Karen G. Haifa, Issam G. Chalboub, Rita T. Boulos, Issam Kassab, Mirna N. Chahine.

**Data curation:** Yara G. Dagher, Sandra El. Helou, Karen G. Haifa, Issam G. Chalboub, Rita T. Boulos, Bachir Atallah, Issam Kassab, Mirna N. Chahine.

**Formal analysis:** Bachir Atallah.

**Investigation:** Yara G. Dagher, Sandra El. Helou, Karen G. Haifa, Issam G. Chalboub, Rita T. Boulos, Issam Kassab, Mirna N. Chahine.

**Methodology:** Bachir Atallah, Issam Kassab, Mirna N. Chahine.

**Project administration:** Issam Kassab, Mirna N. Chahine.

**Resources:** Fadi Nasr.

**Supervision:** Issam Kassab, Mirna N. Chahine.

**Validation:** Fadi Nasr, Mirna N. Chahine.

**Visualization:** Mirna N. Chahine.

**Writing – original draft:** Yara G. Dagher, Sandra El. Helou, Karen G. Haifa.

**Writing – review & editing:** Issam Kassab, Mirna N. Chahine.
